# Small molecule-mediated tribbles homolog 3 promotes bone formation induced by bone morphogenetic protein-2

**DOI:** 10.1038/s41598-017-07932-z

**Published:** 2017-08-08

**Authors:** Jiabing Fan, Joan Pi-Anfruns, Mian Guo, Dan C. S. Im, Zhong-Kai Cui, Soyon Kim, Benjamin M. Wu, Tara L. Aghaloo, Min Lee

**Affiliations:** 1 Division of Advanced Prosthodontics, School of Dentistry, University of California, Los Angeles, California 90095 USA; 2 Division of Diagnostic and Surgical Sciences, School of Dentistry, University of California, Los Angeles, California 90095 USA; 3 Department of Neurosurgery, the 2nd Affiliated Hospital of Harbin Medical University, Harbin, Heilonjiang 150001 China; 4 Department of Bioengineering, University of California, Los Angeles, California 90095 USA

## Abstract

Although bone morphogenetic protein-2 (BMP2) has demonstrated extraordinary potential in bone formation, its clinical applications require supraphysiological milligram-level doses that increase postoperative inflammation and inappropriate adipogenesis, resulting in well-documented life-threatening cervical swelling and cyst-like bone formation. Recent promising alternative biomolecular strategies are toward promoting pro-osteogenic activity of BMP2 while simultaneously suppressing its adverse effects. Here, we demonstrated that small molecular phenamil synergized osteogenesis and bone formation with BMP2 in a rat critical size mandibular defect model. Moreover, we successfully elicited the BMP2 adverse outcomes (i.e. adipogenesis and inflammation) in the mandibular defect by applying high dose BMP2. Phenamil treatment significantly improves the quality of newly formed bone by inhibiting BMP2 induced fatty cyst-like structure and inflammatory soft-tissue swelling. The observed positive phenamil effects were associated with upregulation of tribbles homolog 3 (Trib3) that suppressed adipogenic differentiation and inflammatory responses by negatively regulating PPARγ and NF-κB transcriptional activities. Thus, use of BMP2 along with phenamil stimulation or Trib3 augmentation may be a promising strategy to improve clinical efficacy and safety of current BMP therapeutics.

## Introduction

Mandibular bone defects are commonly caused by traumatic injury, infection, congenital deformity, and secondary treatment of varying pathologies such as tumor resection and drug-induced osteonecrosis, leading to undesirable effects on oral function and appearance^1^. Microvascular free tissue transfer is the preferred approach in current mandibular reconstruction, however, it raises the concern of donor site morbidities and perioperative complications^2, 3^. Tissue engineering approach with osteoinductive growth factors is a promising alternative option for the reconstruction of bony defects especially with large mandibular defects^4, 5^.

Bone morphogenetic protein 2 (BMP2) is believed to be the most potent osteoinductive factor available and has been extensively studied for the treatment of many bone fractures and bone defects^6, 7^. However, the clinical application of BMP2 requires supraphysiological milligram-level doses that may increase inappropriate adipogenesis and cyst-like hollow bone formation^8, 9^. Furthermore, the premature release of such high dose BMP2 from conventional collagen carriers may lead to numerous side effects such as ectopic bone formation, inflammatory soft tissue swelling, or osteoclastic bone resorption^10–12^. Herein, it is necessary to develop an alternative molecular therapeutic approach capable of complementing BMP2 activity to maximized biological efficiency while simultaneously minimizing BMP2-associated adverse effects.

Small molecule phenamil, an amiloride derivative, was shown to effectively invoke the osteogenesis of mesenchymal progenitor cell as well as the enhancement of bone repair in *in vitro* and *in vivo* studies^13–15^. Moreover, our recent studies demonstrated phenamil synergized osteogenesis and bone formation with BMP2 by enhancing BMP/Smad signaling^15, 16^. The increased BMP signaling by phenamil is thought to be through the induction of tribbles homolog 3 (Trib3) that degrades Smad ubiquitin regulatory factor 1 (Smurf1), a negative regulator of BMP receptor-regulated Smads^13, 15, 17^. Moreover, recent studies suggest that Trib3 suppresses the expression of peroxisome proliferator activated receptor gamma (PPARγ), a master regulator of adipogenesis, and serves as a negative regulator of pro-inflammatory cytokines^18–22^. Thus, phenamil treatment has high potential to effectively complement the BMP activity to maximize osteogenesis without exogenous application of supraphysiological BMP doses while inhibiting BMP-induced adverse outcomes (i.e. adipogenesis and inflammation).

Here, we investigated whether phenamil can improve the amount and quality of newly formed bone induced by BMP2. First in this study, we will determine if phenamil can maximize BMP2 induced bone formation in critical-sized large mandibular bone defects created in rats. Next, we will apply high dose BMP2 to induce adverse cyst-like bone formation and inflammation in the mandibular defect model and will test whether phenamil can inhibit BMP2 induced fatty cyst-like structure and inflammatory soft-tissue swelling. Lastly, we will determine whether the positive phenamil effects in the combinatorial treatment of phenamil + BMP2 are through increased Trb3 expression *in vitro* cell culture and *in vivo* early mandibular implantation.

## Results

### Phenamil synergizes bone formation with BMP2 and inhibits BMP2 induced cyst-like bone formation

**Figure 1:**
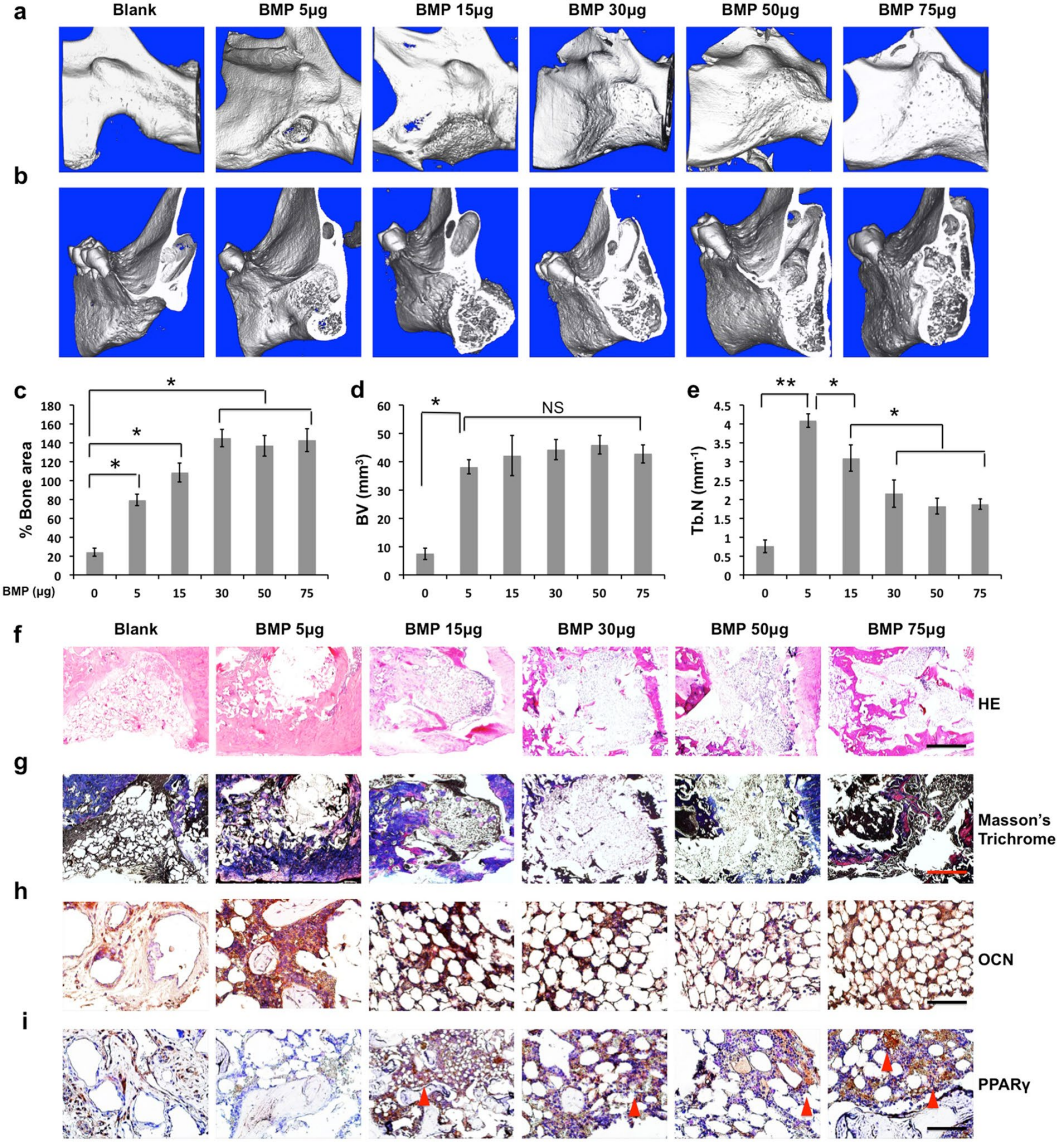
BMP2 dose ranging from 0 to 75 μg (0–1.5 mg/mL) was used to induce cyst-like void bone formation with a dose-dependent fashion in the rat critical-sized (5 × 5 mm) mandibular defect. 8 weeks postoperatively, the collected mandibular implants were measured by the following analysis: Micro-computed tomography (microCT) images in general (**a**) and sagittal sections (**b**); Quantification of % bone surface area (**c**), bone volume (mm^3^) (**d**), and trabecular number (Tb.N, mm^−1^) (**e**); Histological analysis including HE staining (scale bar = 500 µm) (**f**), Masson’s trichrome staining (scale bar = 500 µm) (**g**), and immunohistochemical staining for OCN (scale bar = 50 µm) (**h**) and PPARγ (scale bar = 50 µm) (**i**). Red arrowhead indicates high expression of PPARγ. Blank, blank scaffold; BMP, BMP2; NS, no significant difference. Data presented as means ± SD (n = 3/group); *p < 0.05, **p < 0.01.

Various concentrations of BMP2 (5–75 µg in 50 µl defect volume, 100–1500 µg/ml) were loaded onto apatite-coated PLGA scaffolds and implanted into mandibular defects. MicroCT analysis demonstrated enhanced new bone formation with increasing BMP-2 dose at 8 weeks post-surgery (Fig. 1a and c). Although BMP2 at 30 µg or higher was able to fuse the defects, it also consistently induced hollow cysts and sparse trabecular bone as observed by sagittal microCT (Fig. 1b). The quantitative analysis demonstrated that bone volume was not significantly different among the experimental groups, but trabecular bone number was significantly reduced with increasing BMP-2 dose (Fig. 1d and e). The cystic structure induced by BMP2 at 30 µg or higher presented low osseous matrix production filled with fatty marrow as observed by H&E, Masson’s trichrome, osteocalcin (OCN) and PicroSirius red stain (Fig. 1f–h and Supplementary Fig. 1a). Instead, the extensive expression of adipogenic regulator PPARγ was detected in the bone cysts (Fig. 1i). Moreover, intense TRAP stain was detected in high dose BMP2 treatment over 50 µg, indicating strong osteoclastic bone resorption (Supplementary Fig. 1b).

**Figure 2:**
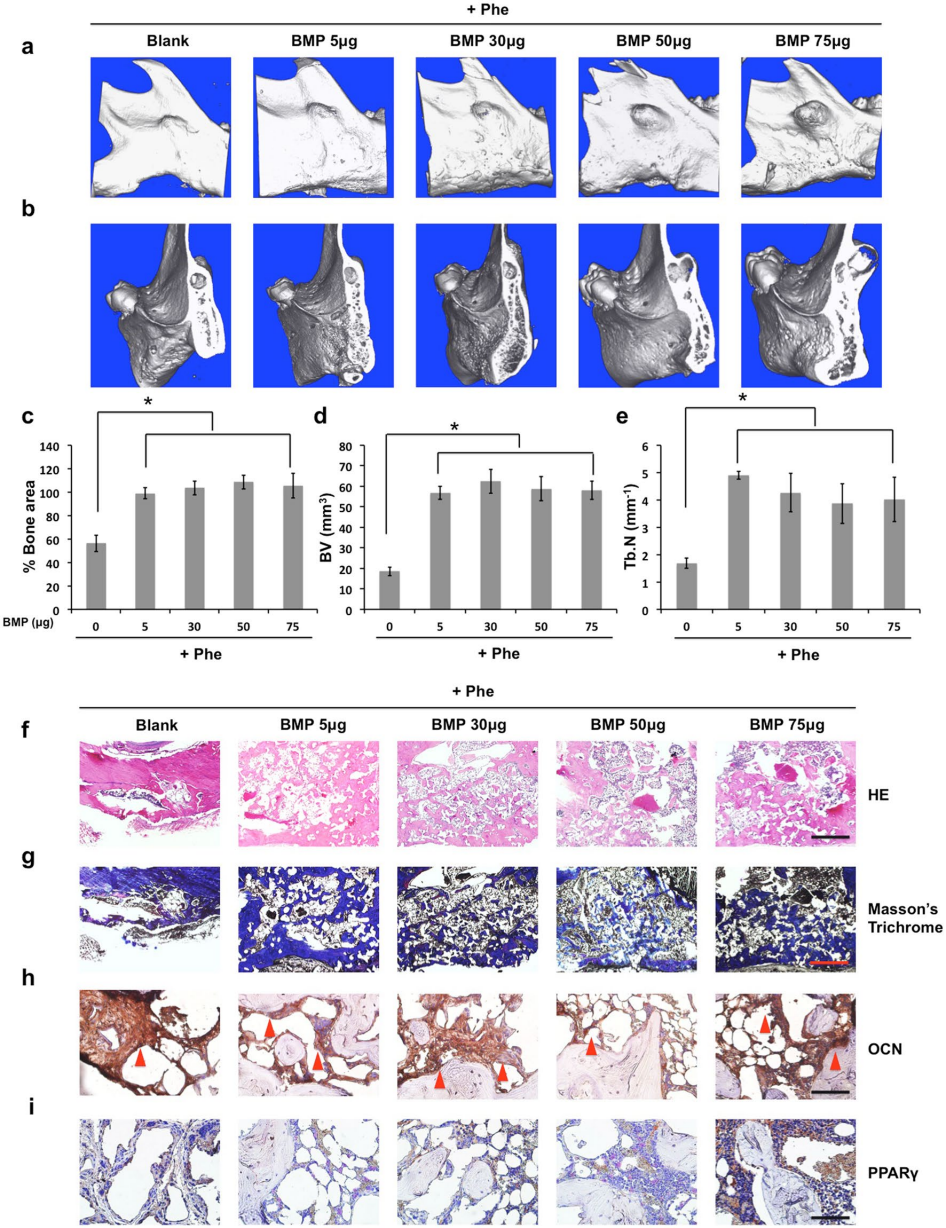
The extra use of phenamil at 600 µM (total 12 μg per defect) impedes high dose BMP2-induced cyst-like void bone formation replaced with new robust ossification formation. 8 weeks postoperatively, the collected implants were measured by the following analysis: Micro-computed tomography (microCT) images in general (**a**) and sagittal sections (**b**); Quantification of % bone surface area (**c**), bone volume (mm^3^) (**d**), and trabecular number (Tb.N, mm^−1^) (**e**); Histological analysis including HE staining (scale bar = 500 µm) (**f**), Masson’s trichrome staining (scale bar = 500 µm) (**g**), and immunohistochemical staining for OCN (scale bar = 50 µm) (**h**) and PPARγ (scale bar = 50 µm) (**i**). Red arrowhead indicates high expression of OCN. Blank, blank scaffold; BMP, BMP2; Phe, phenamil. Data presented as means ± SD (n = 3/group); *p < 0.05.

Next, we investigated efficacy of phenamil to improve BMP2 induced bone formation. Combined treatment with phenamil and BMP2 induced almost complete mandibular bone healing even at very low BMP2 dose (5 µg) (Fig. 2a and c). The combined treatment significantly increased both bone volume and trabecular bone number compared with BMP2 alone group (Fig. 2d and e). Moreover, co-treatment with phenamil + BMP2 resulted in dense trabecular structure without cyst formation at all tested BMP2 doses (Fig. 2b). Histological and immunohistochemical analysis demonstrated conspicuous ossification in all combinatorial treatments (Fig. 2f–h and Supplementary Fig. 2a). In contrast to BMP2 alone groups, minimal expression of PPARγ was observed in phenamil + BMP2 groups (Fig. 2i) with low TRAP positive stain (Supplementary Fig. 2b).

### Phenamil inhibits BMP2-induced inflammatory soft tissue swelling and adipogenesis

**Figure 3:**
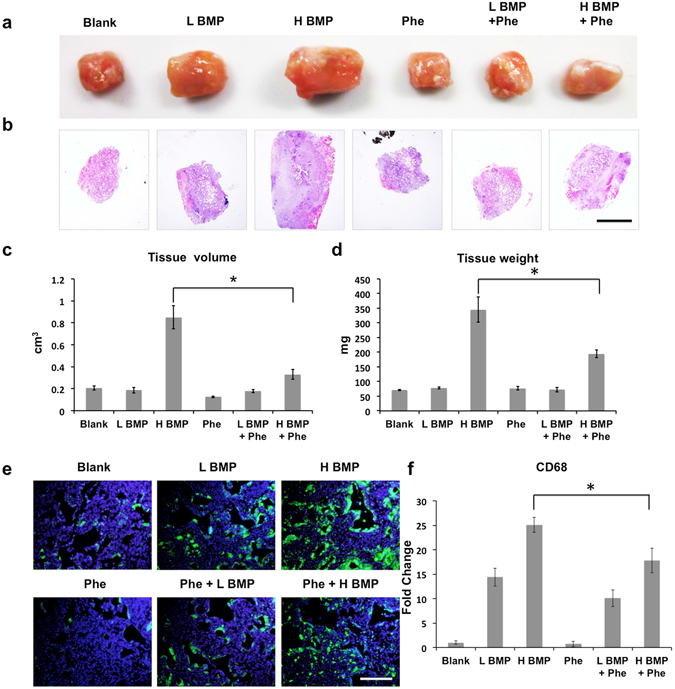
High dose BMP2-induced inflammation/tissue swelling observed in the rat critical-sized mandibular defect as early as 7 days postoperatively was suppressed by the additional treatment of phenamil (600 µM, total 12 μg per defect). Harvested tissue samples from mandibular defect immediately detected by gross image (**a**) and further analyzed with HE staining at low magnification (scale bar = 1.6 mm) (**b**), and also measured by tissue volume (**c**) and tissue weight (**d**), respectively; CD68 expression (representing the presence of macrophage cells) with immunofluorescence staining (scale bar = 200 µm) (**e**) and semi-quantification (**f**). Blank, blank scaffold; L BMP, low dose BMP2 at 5 µg; H BMP, high dose BMP2 at 30 µg; Phe, phenamil at 600 µM. Data presented as means ± SD (n = 3/group); *p < 0.05.

**Figure 4:**
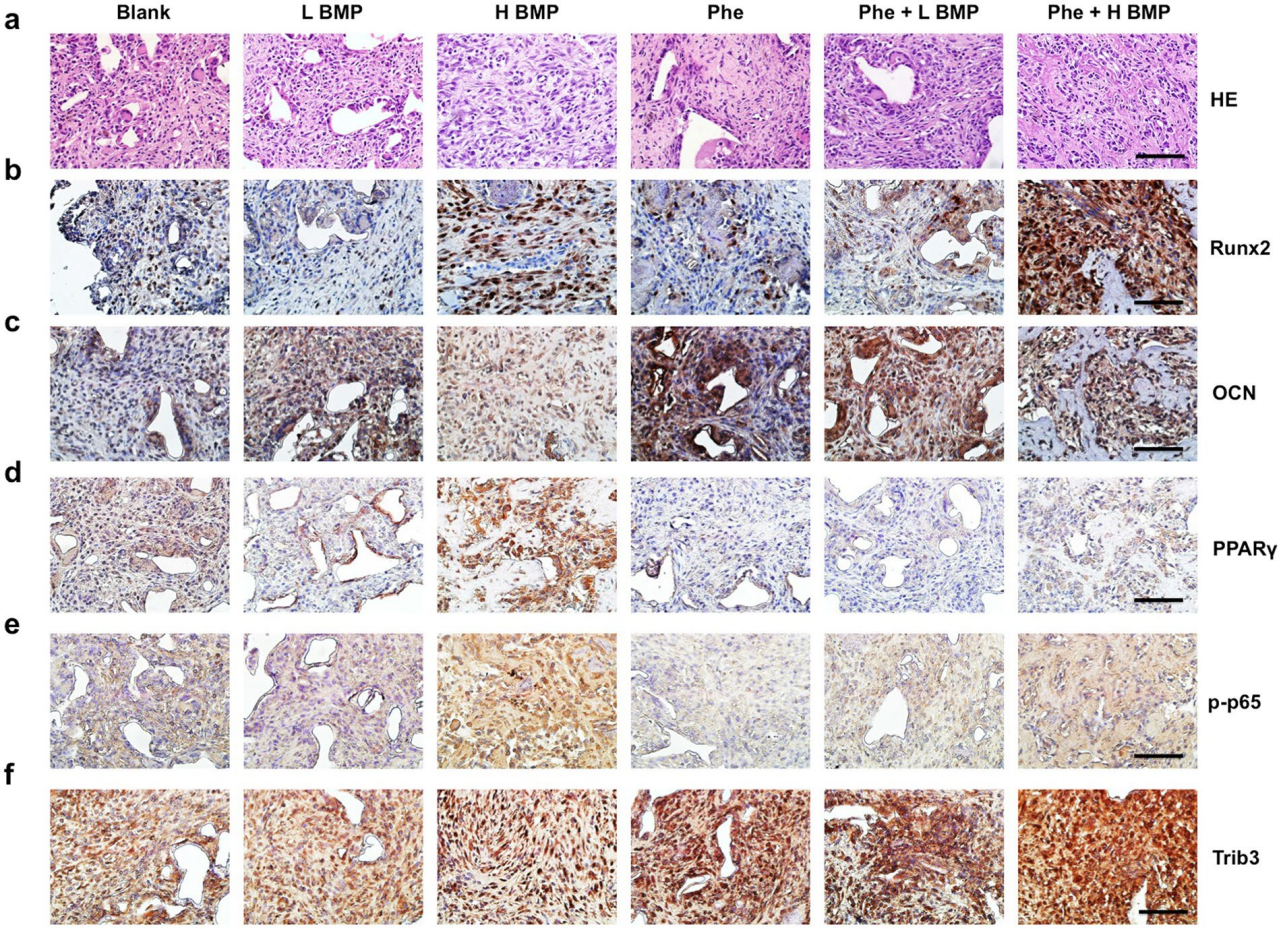
Phenamil-inhibited high dose BMP2-induced inflammation/tissue swelling was analyzed by histological and immunohistochemical analysis. HE staining at high magnification (**a**); Immunohistochemical staining for the detection of Runx2 (**b**), OCN (c), PPARγ (**d**) phosphorylated p65 (**e**), and Trib3 (**f**). Scale bar = 50 µm. Blank, blank scaffold; L BMP, low dose BMP2 at 5 µg; H BMP, high dose BMP2 at 30 µg; Phe, phenamil at 600 µM (total 12 µg per defect); p-p65, phosphorylated-p65.

We further explored the effect of phenamil on BMP2-induced complication in the mandibular defect as early as 7 days postoperatively. Gross and histological observation revealed high tissue swelling in the defect treated with high dose BMP2 (30 μg) (Fig. 3a and b). Quantitative measurement showed that tissue weight and volume were ~8.4- and ~4.5- folds higher in high dose BMP2 groups, respectively, than that of low dose BMP2 (5 μg) groups (Fig. 3c and d). In contrast, high-dose BMP2 groups co-treated with phenamil exhibited no discernable swelling with lower tissue weight and volume (Fig. 3a–d). Moreover, a massive infiltration of cells that positively stain CD68, a macrophage marker, were observed in the implant containing high dose BMP2, but macrophage aggregation was significantly decreased in phenamil + BMP2 groups (Fig. 3e and f). Histological and immunohistochemical analysis showed no apparent neo-bone formation detected in all treated groups by histological analysis (Fig. 4a), while the expression of Runx2 and OCN was significantly increased in phenamil + BMP2 treatment (Fig. 4b and c). Strong staining for PPARγ was detected in high dose BMP2 groups, while the low level of PPARγ expression was observed in phenamil-treated groups with or without BMP2 (Fig. 4d). The expression of phosphorylated p65, a marker of NF-κB activation that regulates inflammatory response, was significantly increased only in implants treated with high-dose BMP2 alone (Fig. 4e). Strong staining for Trib3 was detected in phenamil containing groups, but not in BMP-2 alone groups (Fig. 4f).

### Phenamil inhibits BMP2-induced inflammation *in vitro*

**Figure 5:**
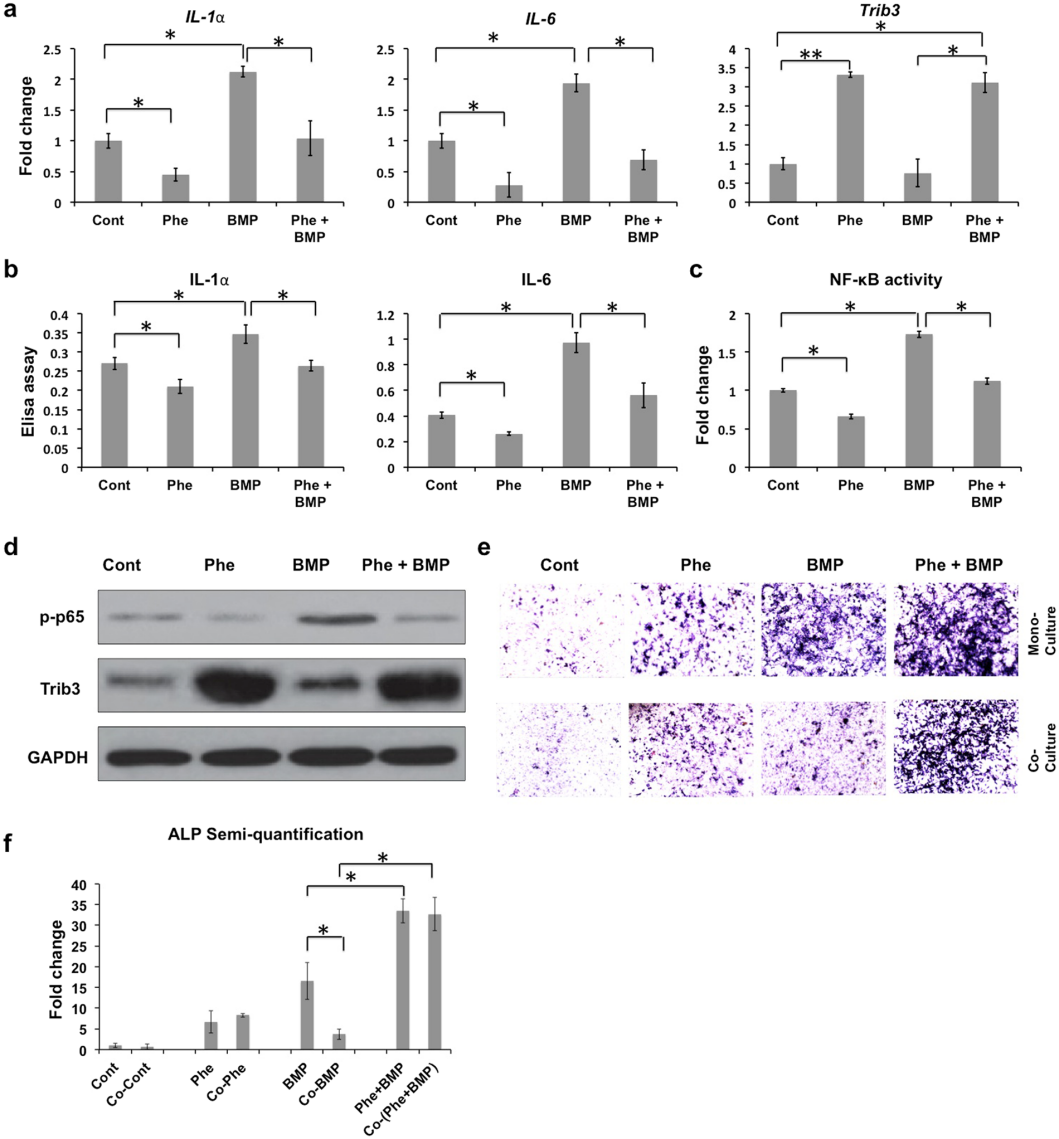
*In vitro* analysis of the effects of phenamil treatment on high doses BMP2-induced inflammation in RAW264.7 cells. (**a**) Increased inflammatory genes (*IL-1*α and *IL-6*) in BMP2 (500 ng/mL) treatment were suppressed by extra phenamil treatment (20 µM) *via* the increase of *Trib3*. The level of gene expression was measured by real-time PCR assay; (**b**) Elisa assay was utilized to detect inflammatory cytokines expression (IL-1α and IL-6); (**c**) NF-κB activities was examined by luciferase assay; (**d**) Western-blot assay confirmed phenamil-mediated inflammatory prohibition by increased Trib3 expression to suppress phosphorylated p65 (NF-κB signaling); (**e**,**f**) A co-culture system between RAW264.7 and BMSCs showed that phenamil rescued the high-dose BMP2-mediated inhibitory osteogenesis by ALP staining (**e**) and semi-quantification (**f**). Scale bar = 200 µm. Ctr, Control; Phe, phenamil; BMP, BMP2; p-p65, phosphorylated-p65. Data presented as means ± SD (n = 3/group); *p < 0.05, **p < 0.01.Uncropped western blot gels are displayed in Supplementary Figure 6.

To investigate whether phenamil can inhibit BMP2-induced inflammation *in vitro*, RAW264.7 cells (a macrophage cell line) were treated with BMP2 at 500 ng/mL in the presence or absence of phenamil. The results demonstrated that BMP2 stimulation significantly increases expression of *IL-1*α and *IL-6* compared with control (Fig. 5a). Addition of phenamil reversed their expression to the level comparable with control (Fig. 5a). Consistently, ELISA assay indicated that phenamil treatment suppressed the secretion of inflammatory cytokines (IL-1α and IL-6) in the presence or absence of BMP2 (Fig. 5b). The observed decrease in inflammatory reaction was accompanied by upregulation of Trib3 (Fig. 5a). The similar anti-inflammatory effects of phenamil were confirmed in the NIH3T3 fibroblast and C2C12 myoblast cell lines (Supplementary Fig. 3a and b). Since the expression of inflammatory cytokines may depend on NF-κB activity, we further monitor the activity of NF-κB signaling pathway in RAW264.7 by luciferase NF-κB reporter assay. The assay measured significant increase in NF-κB transcription activity by BMP2. In contrast, the BMP-driven promoter activity was decreased after phenamil treatment (Fig. 5c). Moreover, western-blot assay demonstrated that phenamil impedes BMP2-induced phosphorylation of p65 and this was accompanied by elevation of Trib3 level (Fig. 5d, Supplementary Fig. 6). We further investigated the effects of phenamil-mediated anti-inflammation in BMP2 induced osteogenesis by co-culturing BMSCs with RAW 264.7 cells with phenamil (20 μM) ± BMP2 (500 ng/mL). ALP expression of BMSCs induced by BMP2 was significantly reduced by co-culture with RAW 264.7 cells (Fig. 5e and f). In contrast, RAW 264.7 cells did not change the level of ALP expression in BMSCs in the presence of phenamil (Fig. 5e and f).

### Knockdown of Trb3 attenuates pro-osteogenic and anti-adipogenic activities of phenamil *in vitro*

**Figure 6:**
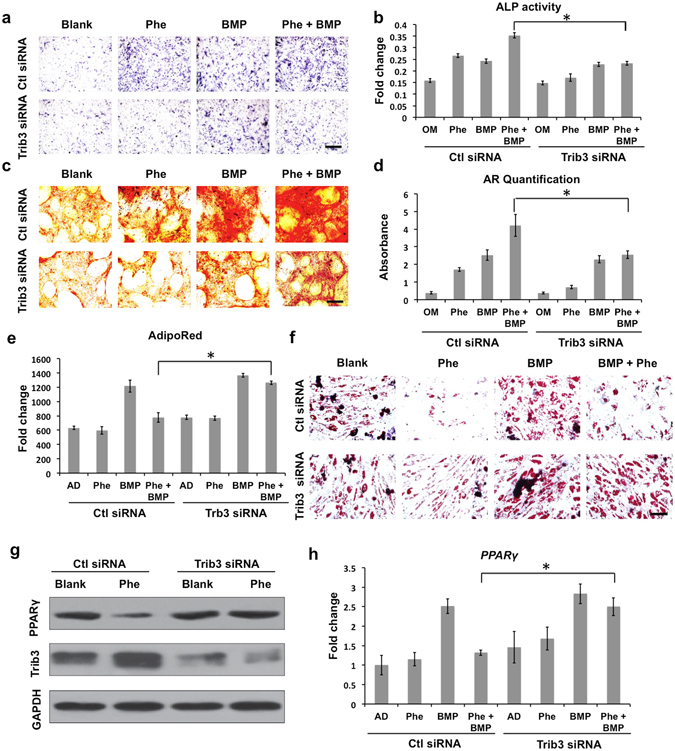
The role of Trib3 induced by phenamil in the BMP2-mediated osteogenesis and pro-adipogenesis by examining Trib3 knockdown hBMSCs. (**a**–**d**) Blocking Trib3 expression led to the loss of synergistic effect of phenamil (20 µM) on BMP2 (100 ng/mL)-based osteogenesis by the detection of ALP staining/activity (scale bar = 200 µm) (**a**,**b**), and Alizarin red S staining/quantification (scale bar = 100 µm) (**c**,**d**); (**e**,**f**) Down-regulated Trib3 attenuated the anti-adipogenic capacity of phenamil (10 µM) on high dose BMP2 (300 ng/mL)-induced adipogenesis *via* Adipored assay at day 7 (**e**) and Oil red staining (scale bar = 100 µm) at day 14 (**f**); (**g**) Phenamil exerted anti-adipogenesis *via* up-regulating Trb3 that inhibited PPARγ expression. (**h**) Trib3 knockdown attenuated phenamil (10 μM)-mediated inhibitory BMP2 (300 ng/mL)-induced adipogenic genes: PPARγ in hBMSCs. Phe, phenamil; BMP, BMP2; OM, osteogenic medium; AD, adipogenic medium. Data presented as means ± SD (n = 3/group); *p < 0.05. Uncropped western blot gels are displayed in Supplementary Figure 6.

We investigated the role of Trib3 in phenamil + BMP2 mediated mesenchymal stem cell differentiation *in vitro*. hBMSCs were transduced with Trib3 siRNA or control siRNA and treated with phenamil (20 μM) and/or BMP2 (100 ng/mL). Treatment of hBMSCs with phenamil significantly increased ALP activity and mineralization induced by BMP2 (Fig. 6a–d). In contrast, knockdown of Trib3 significantly reduced phenamil-induced osteogenic differentiation of hBMSCs in the presence or absence of BMP2 (Fig. 6a–d). Phenamil was shown to upregulate *Trib3* expression and osteogenic differentiation markers including *Runx2*, *ALP* and *OCN* in the presence or absence of BMP2, but knockdown of Trb3 significantly reduced the expression of osteogenic gene markers induced by phenamil ± BMP2 (Supplementary Fig. 4a–d). Phenamil-induced Trib3 expression was further confirmed by western blot and the increased Trib3 downregulated Smurf1, a negative regulator of Smads, and thereby enhanced levels of Smads (Supplementary Figs 4e and 6). By contrast, Trib3 knockdown suppressed phenamil-induced BMP/Smad signaling (Supplementary Figs 4e and 6).

We further investigated whether phenamil can inhibit BMP2 induced adipogenic differentiation. BMP2 treatment (300 ng/mL) increased lipid accumulation in hBMSC, while the addition of phenamil (10 μM) significantly reduced BMP2 induced adipogenesis as observed by Adipored assay (Fig. 6e) and Oil Red O staining (Fig. 6f). In contrast, knockdown of Trib3 abrogated the observed effects of phenamil to inhibit adipogenesis. Phenamil upregulated *Trib3* expression and suppressed expression of adipogenic regulatory genes *PPARγ* and *LPL* induced by BMP2, while knockdown of Trib3 reversed the anti-adipogenic effect of phenamil (Fig. 6h and Supplementary Fig. 5a,b). Phenamil inhibition of PPARγ activity was further confirmed by western blot and the inhibitory effect was accompanied by enhanced Trib3 level (Fig. 6g and Supplementary Fig. 6). In contrast, Trib3 knockdown reversed the phenamil-mediated PPARγ suppression (Fig. 6g).

## Discussion

Food and Drug Administration (FDA)-approved BMP2 has demonstrated extraordinary potential in bone formation and is widely used in skeletal repair including spinal fusion, tibial fracture repair, and alveolar ridge/maxillary sinus augmentation, however its supraphysiological dose requirement has revealed numerous adverse outcomes, such as excessive osteoclastogenesis with vertebral osteolysis and life-threating inflammatory cervical swelling^23–26^. In addition, high BMP dose increases PPARγ activity and adipogenesis, resulting in cyst-like void bone formation^8, 9^. Previous reports and our recent study demonstrated that small molecular phenamil synergistically induced osteogenesis and bone formation with BMP2 through upregulation of Trib3, a positive regulator of BMP signaling, while lowering BMP2 dose requirement without compromising osteogenic efficacy^13, 15, 16^.

Tribbles homologs family members (Trib) exert diverse roles in development, cellular differentiation, and inflammation^18^. Among Trib isoforms, Trib3 has been most extensively studied^21, 22, 27^ and recent study indicates its critical role in modulating BMP pathway by binding to Smurf1^13, 17^. In response to BMP stimulation, Trib3 is dissociated from BMP receptor and degrades Smurf1, stabilizing Smads and potentiating BMP signaling^17^. Downregulation of Trib3 inhibited BMP-mediated osteogenic differentiation of mesenchymal stem cells (MSCs). In addition to its critical role in osteogenic differentiation, recent studies also indicate that Trib3 regulates adipogenesis and inflammation by interacting with numerous transcription factors such as PPARγ, CCAAT/enhancer-binding protein beta (C/EBPB), and NF-κB^19–22^. Therefore, Trib3 may be a promising molecular target to enhance BMP2 induced osteogenesis and reduce adverse effects.

In this study, we increased Trib3 expression using phenamil, a small molecule inducer of Trib3, and evaluated the synergistic ability of phenamil with BMP2 to promote bone regeneration in large mandibular discontinuity defects. Phenamil has been shown to stimulate BMP signaling and osteogenesis by upregulating Trib3 expression in our previous *in vitro* and *in vivo* studies^15^. Our data showed that the addition of phenamil to BMP-2-treated defects significantly increased bone regeneration over defects treated with BMP-2 alone, and the observed increase in bone healing by phenamil was induced through Trib3 upregulation in the defects. While the molecular mechanism is not fully understood, the amiloride analog phenamil is known to inhibit ion channel function in cells, which could be critical for Trib3 induction. Previous study demonstrated that phenamil increased Trib3 transcriptional activity via activation of the calcium-calcineurin-unclear factor of activated T cell (NFAT) pathway and this was mediated by inhibition of acid-sensing ion channels (ASICs)^28^.

Since BMP2 adverse outcomes are dose-dependent, we applied various concentrations of BMP2 up to human dose equivalent BMP2 (75 µg per defect, 1.5 mg/ml) in our rodent mandibular defect model to elicit adverse outcomes associated with high dose BMP2 such as adipogenic differentiation with cyst-like bone formation. Although new bone formation was accelerated with increasing BMP2 dose, BMP2 at 30 µg (600 µg/ml) or higher consistently induced hollow cysts filled with fatty marrow and distinct PPARγ. Similar observation of BMP2-induced structurally abnormal bone has been reported in a rat long bone defect model^29^. In contrast, co-treatment with phenamil + BMP2 resulted in dense trabecular structure without cyst formation. We also confirmed strong expression of Trib3 in phenamil-treated groups. Since phenamil dose-dependently increased osteogenic differentiation in MSCs up to 10–20 μM and bone formation up to 300–600 μM in our previous study^15^, a similar concentration of phenamil at 20 μM and 600 μM was tested in the present *in vitro* and *in vivo* studies respectively. However, the obtained results may not predict the most optimal phenamil dose and additional studies will be required to investigate the dose-dependent effect of phenamil.

Cervical swelling is the well-documented severe inflammatory complication associated with BMP2^29, 30^. The elevated levels of pro-inflammatory cytokines were detected in seroma in posterior cervical fusion after BMP2 treatment^30–32^. Excessive local inflammation is well known to delay bone regeneration processes^33, 34^. Gross observation of the mandible in high BMP2 at day 7 revealed high tissue swelling with a massive infiltration of inflammatory cells and p65 positive cells, indicating a strong inflammatory response and NF-κB activation. The observed BMP2 induced inflammation was significantly suppressed by phenamil treatment and this was accompanied by upregulation of Trib3.

We further investigated the role of Trib3 in phenamil + BMP2 induced inflammation and mesenchymal cell differentiation *in vitro* by culturing RAW264.7 macrophage cells and BMSCs with BMP-2 in the presence of absence of phenamil. When cultured with high dose BMP2, RAW 264.7 cells increased NF-κB transcription activity and cytokine expression, which were significantly suppressed with phenamil treatment. This anti-inflammatory effect of phenamil was accompanied by upregulation of Trib3. Trib3 was well documented to be a negative regulator of NF-κB signaling that closely correlates with inflammatory response and immune response^33–35^. Furthermore, osteogenic differentiation of BMSCs induced by BMP2 was significantly reduced by co-culturing RAW264.7 cells but their osteogenic phenotype was rescued with the additional of phenamil, indicating the positive effect of phenamil-inhibited inflammatory cytokine secretion on osteogenesis. Increasing evidences demonstrated that over-activation of NF-κB by inflammatory cytokine such as IL-1 and TNF-α may impair the osteogenic differentiation of progenitor cells and bone formation^33, 34^. The similar inhibitory effect of inflammation was reported in mesenchymal precursor cells treated with BMP-2 and the observed inhibitory effect was reversed by a blocking of IL-6 expression or NF-κB signaling^36, 37^. Further study of using specific inhibitors will be needed to determine whether the adverse high-dose BMP effects or the positive phenamil effects are dependent on inflammatory activities with the co-culture system.

Additionally, the balance of osteogenesis and adipogenesis is crucial in bone remolding during bone homeostasis^38^. Growing evidences suggested that shifting of MSCs differentiation toward adipogenesis is associated with fatty marrow accretion closely linked to age-related bone disorder like osteoporosis^39^. In the regimen of bone tissue engineering, high dose BMP2 required for clinical bone repair may increase PPARγ activity and adipogenesis resulting in undesired cyst-like bone formation filled with lipid^28^. Our *in vitro* results showed that phenamil significantly reduced BMP2 induced adipogenesis of MSCs. This effect was confirmed by expression of PPARγ and was mediated by upregulating Trib3. In contrast, knockdown of Trb3 abrogated the observed effects of phenamil to inhibit adipogenesis, suggesting that the anti-adipogenic effect of phenamil may be via Trib3 activation. Taken together, our complementary strategy of BMP2 treatment along with phenamil-mediated Trib3 expression could improve the efficacy and safety of BMP2 in new bone formation by enhancing BMP2 induced osteogenesis and reducing adverse BMP2 outcomes. The additional knowledge gained from this study may suggest therapeutic strategies that favor osteoblastogenesis over adipogenesis for improved skeletal regeneration and prevention of osteoporotic fractures.

## Conclusions

Our study demonstrated that phenamil effectively enhanced BMP2 induced osteogenesis and prevented clinically relevant adverse effects associated with BMP2 therapy such as adipogenesis and inflammation in a rat mandibular defect model. This was accompanied by upregulation of Trib3 and replicated finding in mesenchymal cell culture was reversed after knockdown of Trib3, suggesting important roles of Trib3 in phenamil + BMP2 induced osteogenesis. Thus, augmentation of Trib3 expression may be a promising therapeutic strategy to improve the quality of BMP2 induced bone formation.

## Materials and Methods

### Animal care

All experiments were conducted in compliance with institutional guidelines established by the Chancellor’s Animal Research Committee of the Office for Protection of Research Subjects at the University of California, Los Angeles (UCLA) and the UCLA Office of Animal Research Oversight. All experimental protocols were approved by the UCLA Animal Research Committee (Approval #2008–073 and #2003–093). A total of 102 male Sprague Dawley rats at age of 8–12 weeks were purchased from Charles River. All animals were cared in compliance with Guidelines for the Care and Use of Laboratory Animal of the National Institutes of Health. Rats were housed at 2 animals per cage on a 12 hours light/dark cycle with a temperature and humidity-controlled room. All animals were fed standard Teklad Rodent Diets, and each of them had *ad libitum* access to water and food.

### Mandibular defect model

The critical-size 5 × 5 mm mandibular defects were created in rats by the surgical procedures as previously developed^40^. Briefly, the rats were subjected to general anesthesia with isoflurane gas, shaved on the ventral surface of mandible, and subsequently prepped and draped in a sterile manner. An incision overlying and parallel to the left mandible was made using a #15 blade. The inferior border of the mandible was then identified underneath the subcutaneous tissues. After isolating the pterygomasseteric sling with an electrocautery, the lingual and buccal surfaces of the mandibular body were exposed with further supraperiosteal lifting of the musculature. A 1-mm high-speed cutting burr (3,000 RPM) was then used to drill a 5 × 5 mm defect with constant copious irrigation. An equivalent size of apatite-coated PLGA scaffold loaded with drugs (BMP2 purchased from R&D system, MN and GenScript, NJ) was instantly placed onto the mandibular defect using a resorbable suture, followed by a skin closure with non-resorbable suture. All rats after surgery were allowed to recover from anesthesia on warm sheets and then transported to the vivarium for postoperative care. Postoperatively, all rats received analgesia via subcutaneous injections of buprenorphine (0.1 mg/kg) up to 3 days. 6 rats per group were set up for implantation.

### Micro-computerized tomography (µCT) scanning

Animals were euthanized after 8 weeks of post-operation. Left hemi-mandible tissues were collected and instantly fixed in 4% formaldehyde with gentle shaking at room temperature for 48 hours. The fixed tissue samples were then rinsed with PBS solution three times and stored in 70% ethanol prior to imaging using a high-resolution microCT machine (µCT SkyScan 1172; SkyScan, Kontich, Belgium) at 10 µm resolution, 57 kVp, 184 µA and 0.5 mm aluminum filtration. All images were visualized and reconstructed using Dolphin 3D software (Dolphin Imaging & Management Solutions, Chatsworth, CA). The volume and area of new bone were assessed using CTAn (SkyScan, Kontich, Belgium) and Image J software (NIH), respectively. Bone-specific analysis included: new bone area/original defect area (% area) and new bone volume (BV, mm^3^), trabecular number (Tb.N, mm^−1^).

### Histological, immunohistochemical and immunofluorescence analyses

The mandibular implants were extracted from sacrificed rats at week 1 postoperatively, and their volume and weight were measured immediately. For further histological and immunohistochemical analysis, the extracted mandible tissues from 1 weeks and 8 weeks post-operation were fixed with 10% formalin over 2 days. Subsequently, the fixed mandible tissues were decalcified by immersing them into 10% ethylenediaminetetraacetic acid (EDTA) solution with gentle shaking for two weeks, embedded in paraffin and sliced into 5 µm thickness of sections. The tissue sections were then deparaffinized with xylene (Sigma), hydrated in a gradient of ethanol and stained with hematoxylin and eosin (H&E) solution. The deparaffinized sections were further stained with 0.1% Picrosirius red solution (Polysciences, Inc., PA) for detection of collagen expression and were visualized under polarizing light microscope. Masson’s trichrome staining (Sigma) and tartrate-resistant acid phosphatase (TRAP) kit (Sigma) were adopted to detect new bone formation marked with light blue color, and osteoclast cell labeled with purple color respectively. In addition to histological staining, the tissue sections were also subjected to the immunohistochemical analysis. The deparaffinized sections underwent citric acid antigen retrieval, were incubated with the primary antibodies (Santa Cruz) of anti-Trib3, PPARγ, NF-kB p65 (phospho), Runx-2, or OCN and stained by HRP/DAB detection kit (Abcam, MA) following the manufacturer’s instructions. The sections were also counterstained with Mayers’s Hematoxylin (Abcam), and visualized under microscope. At 7 days postoperatively, the fixed tissue sections additionally underwent the immunofluorescence staining on CD68 expression by sequential incubation with primary antibodies of anti-CD68 and fluorochrome-conjugated secondary antibody along with DAPI staining for distinguishing cell nucleic acid. Each immunofluorescence image was further quantified by image J software (NIH).

### Cell culture and transduction

Human bone marrow mesenchymal stem cells (hBMSCs) (n = 2 patients) purchased from Lonza (Vancouver, Canada), were amplified in human MesenCult™ proliferation medium (STEMCELL Technologies, Vancouver) to passage 2 for experiments. Mouse bone marrow stromal cells (mBMSCs, ATCC, VA), RAW264.7, NIH3T3, and C2C12 cells were proliferated under the growth medium (Invitrogen, CA) containing Low Glucose Dulbecco’s Modified Eagle Medium (LDMEM) and 10% fetal bovine serum (FBS). siRNA vectors targeting Trib3 or control (Santa Cruz Biotechnology, Inc., CA) were transduced to hBMSCs following the manufacturer’s protocols. In general, siRNAs at a concentration of 10 nM was transfected into cells at 70% confluence on the 12-well cell culture plate by using Lipofectamine RNAiMAX (Invitrogen).

### Alkaline phosphatase (ALP) activity, Alizarin red staining, Adipored assay and Oil red staining

To measure the degree of osteogenesis, the transduced hBMSCs on the 12-well cell culture plate were grown in the culture medium until approximately 100% confluence, and then the growth medium was replaced with osteogenic medium (Sigma, MO) containing 10 mM β-glycerophosphate, 50 µg/mL L-ascorbic acid, and 100 nM dexamethasone, supplemented with and without phenamil (20 μM) (Sigma) and/or BMP-2 (100 ng/ml) (R&D System, MN). At day 3 of osteoinduction, the cells were fixed with 10% formalin (Sigma), stained with ALP colorimetric assay kit (Sigma) containing Nitro Blue tetrazolium, 5-Bromo-4-chloro-3-indoxylphosphate and AP buffer (100 mM Tris pH 8.5, 100 mM NaCl, 50 mM MgCl_2_), and imaged with Olympus BX 51 microscope (Japan). To further assess ALP activity, the cells were digested in 0.2% NP-40 lysis buffer (Life technologies, CA), subsequently incubated in the buffer containing p-nitrophenol phosphate substrate (Sigma), and measured of the absorbance at 405 nm using a multi-plate reader. Each measurement was finally standardized with total DNA content detected by PicoGreen dsDNA Assay (Life technologies). Alizarin red staining was performed to detect calcium deposition of cells after 2 weeks of osteogenic induction. The fixed cells with 10% formalin, were then stained with 2% alizarin red solution (Sigma) for 5 mins, and visualized with an Olympus BX 51 microscope. The stained cells were further quantitatively analyzed through dissolving the stained cells in 10% (v/v) acetic acid followed by measurement of the absorbance at 405 nm.

To analyze adipogenic differentiation, the transduced cells in 12-well plate at approximately 100% confluence were incubated in human MesenCult™ adipogenic medium (Lonza). Intracellular lipid accumulation within the differentiated adipocytes was assessed after 1 week of post-induction using the Adipored kit (Lonza) by absorbance at 572 nm. For visualization of extracellular lipid expression, the cells at week 2 post-induction were stained with Oil Red O solution (Sigma) for 15 mins and were imaged with the microscope.

### RNA extraction and quantitative real-time polymerase chain reaction (qRT-PCR)

Total RNA extraction from experimental cells was conducted using Trizol reagent (Life Technologies) and RNeasy Mini kit (Qiagen, CA) following the manufacture’s protocol. 500 ng aliquot of RNA for each sample was used to synthesize complementary DNA via a SuperScript III First-Strand Synthesis System (Life Technologies) according to the manufacturer’s instructions. Quantitative real-time PCR analysis was carried out using a 20 µL of SYBR Green reaction system in a LightCycler 480 PCR instrument (Roche, IN). *GAPDH* expression level was measured to normalize the expression level of each target gene. The sequences of each primer were listed in Supplementary Table 1.

### Luciferase Assay

pNF-κB luciferase reporter assay was conducted following the manufacturer’s instructions. In brief, when the RAW264.7 cells on a 24-well cell culture plate reached approximately 70% confluence, they were co-transfected with pNF-κB and control plasmids (Promega, WI) using lipofectamine 2000 (Life Technologies). After 6 hours of transfection, the medium was replaced with cell growth medium supplemented with or without phenamil (20 μM) and/or BMP-2 (500 ng/mL). After extra 48 hours of treatment, luciferase activities of cells were measured by a dual luciferase reporter system (Promega), followed by normalization with Renilla internal control.

### Western-blot assay

The western blot assay was performed according to the protocol previously established^15^. Briefly, the cells were lysed in 0.2% NP-40 lysis buffer (Life technology), followed by the measurement of protein concentration using a bicinchoninic acid protein assay (Thermo Scientific, IL). The protein lysates of cells were then separated by Sodium Dodecyl Sulfate-Polyacrylamide Gel Electrophoresis (SDS-PAGE), blotted onto Immobilon polyvinyl difluoride (PVDF) membrane (Millipore, Billerica, MA), and incubated in the primary antibodies containing anti-Trb3 (Santa Cruz), anti-PPARγ (Santa Cruz), anti-Smurf1, anti-pSmad1/5/8 (Santa Cruz), anti-NF-kB p65 (phospho) or anti-GAPDH (Santa Cruz). The membranes were further incubated in Horseradish Peroxidase (HRP)-conjugated secondary antibody (Millipore, MA), subsequently visualized with chemiluminescent HRP (Denville Scientific, NJ).

### Co-culture of cells

Transwell Permeable Supports (3 μm porous) (Corning, NY) were applied to paracrine non-contact cell co-cultures following the manufacturer’s guidance. Briefly, the RAW264.7 cells and growth medium were added in the 12-well cell culture plate, subsequently Transwell support was inserted into each well onto which BMSCs and growth mediums were added. After 24 hours of co-culture, the growth medium was replaced with low serum (2% FBS) culture medium in the presence or absence of BMP-2 (500 ng/ml) and/or phenamil (20 μM). Over 7 days of *in-vitro* culture, the BMSCs growing in the Transwell support under osteogenic medium were stained by ALP assay. In addition, the mono-cultured medium only in RAW264.7 cells at day 3 was collected for the examination of inflammatory cytokines secretion through a multi-analyte ELISArray kit (GIAGEN, Hilden, Germany) according to manufacturer’s instructions.

### Scaffold fabrication

The apatite-coated PLGA scaffold was synthesized through the process of solvent casting and particulate leaching methodology as previously established^15^. In general, PLGA/Chloroform mixed solution was blended with 200–300 µm diameter of sucrose to form a porosity of approximate 92% (volume fraction), compressed into a Teflon mold and freeze-dried overnight at −110 °C and 100 mTorr (SP Industries, Inc., PA). Subsequently, the scaffolds were plunged in double-distilled (dd) H_2_O to remove sucrose and then sterilized in 70% ethanol up to 30 mins, followed by several rinses in sterile ddH_2_O. And then the dried scaffold sheets were sliced into small plates at the size of 5 × 5 × 2 mm. Finally, the PLGA scaffold plate was coated an apatite layer through incubating it in simulated body fluid (SBF): The scaffold was first subjected to glow discharge argon plasma etching (Harrick Scientific, Pleasantville, NY); The etched scaffold was then incubated in SBF1 formulated with the mixture of CaCl_2_, MgCl_2 _· 6H_2_O, NaHCO_3_, K_2_HPO_4 _· 3H_2_O, Na_2_SO_4_, KCl and NaCl for 24 hours and subsequently in SBF2 with the mixture of CaCl_2_, K_2_HPO_4 _· 3H_2_O, KCl and NaCl for another 24 hours at 37 °C.

### Statistical analysis

Statistical analysis in the studies was performed by one-way analysis of variance (ANOVA) with the Tukey’s post hoc test. The data were presented as means ± SD. P-value < 0.05 was considered statistically significant.

## Electronic supplementary material

Supplementary data
