# Supplementary material for: Small molecule-mediated tribbles homolog 3 promotes bone formation induced by bone morphogenetic protein-2
Source: Sci Rep. 2017 Aug 8;7:7518. doi: 10.1038/s41598-017-07932-z (PMC5548928; doi:10.1038/s41598-017-07932-z)
Supplement: Supplementary file 1 — Supplementary data [file 41598_2017_7932_MOESM1_ESM.pdf]

Small molecule-mediated tribbles homolog 3 promotes bone formation induced by bone morphogenetic protein-2

Jiabing Fan[1], Joan Pi-Anfruns[2], Mian Guo[3], Dan C.S. Im[1], Zhong-Kai Cui[1], Soyon Kim[4], Benjamin M. Wu[1,4], Tara L. Aghaloo[2,*], Min Lee[1,4,*]

[1]Division of Advanced Prosthodontics, School of Dentistry, University of California, Los Angeles, California 90095
[2]Division of Diagnostic and Surgical Sciences, School of Dentistry, University of California, Los Angeles, California 90095
[3]Department of Neurosurgery, the 2nd Affiliated Hospital of Harbin Medical University, Harbin, Heilonjiang, China 150001
[4]Department of Bioengineering, University of California, Los Angeles, California 90095

[*]Co-senior authors

Corresponding authors: Min Lee, PhD
Division of Advanced Prosthodontics
UCLA School of Dentistry
10833 Le Conte Avenue, CHS 23-088F
Los Angeles, CA 90095-1668
Telephone: (310) 825-6674
Fax: (310) 825-6345
Email: leemin@ucla.edu

Tara L. Aghaloo, DDS, MD, PhD
Division of Diagnostic and Surgical Sciences,
UCLA School of Dentistry
10833 Le Conte Ave, CHS 53-009,
Los Angeles, CA 90095-1668
Telephone: (310) 206-6766
Fax: (310) 825-7232
Email: taghaloo@dentistry.ucla.edu

Supplementary Figures:

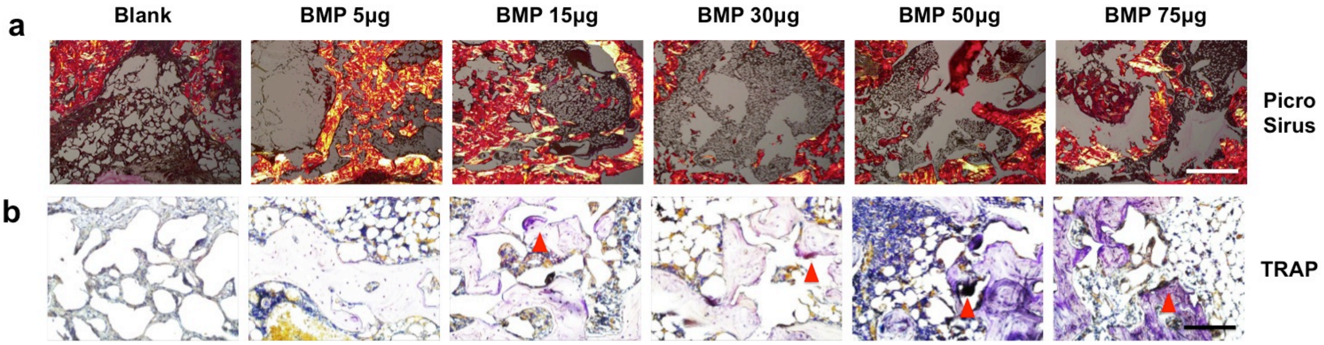

Supplementary Figure 1: High doses BMP2-induced cyst-like hollow bone formation detected in the rat mandible defect after 8 weeks postoperatively. (a) Picrosirius red staining with polarized images (scale bar = 500 μm); (b) TRAP staining for identifying osteoclasts (scale bar = 100 μm). Red arrowhead indicates osteoclasts along the edge of bone. Blank, blank scaffold; BMP, BMP2; TRAP, tartrate-resistant acid phosphatase.

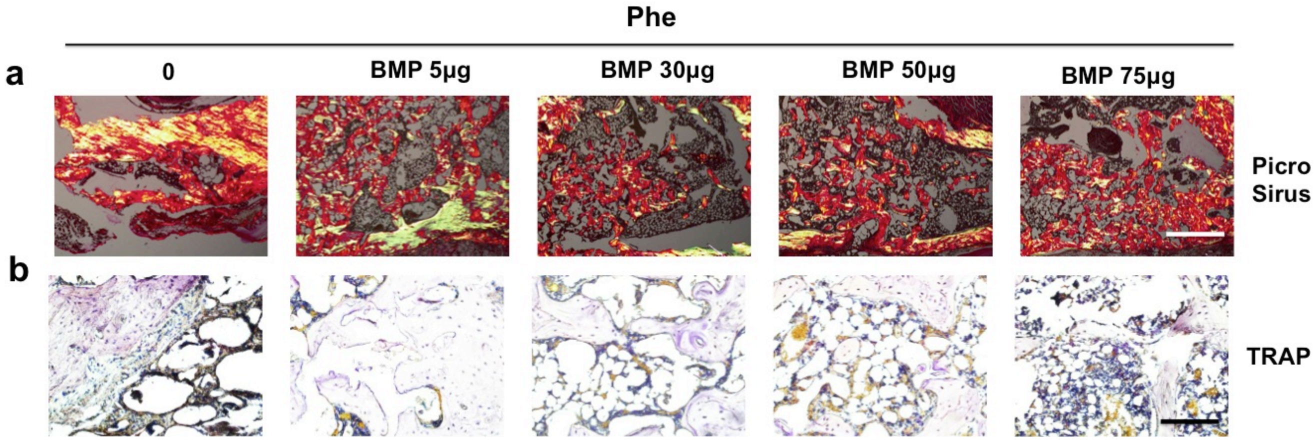

Supplementary Figure 2: Extra use of phenamil (600 μM, total 12 μg per defect) suppressed high doses BMP2-induced cyst-like hollow bone formation in the rat mandible defect after 8 weeks postoperatively. (a) Picrosirus red staining with polarized images (scale bar = 500 μm); (b) TRAP staining for identifying osteoclasts (scale bar = 100 μm). Blank, blank scaffold; BMP, BMP2; Phe, phenamil; TRAP, tartrate-resistant acid phosphatase.

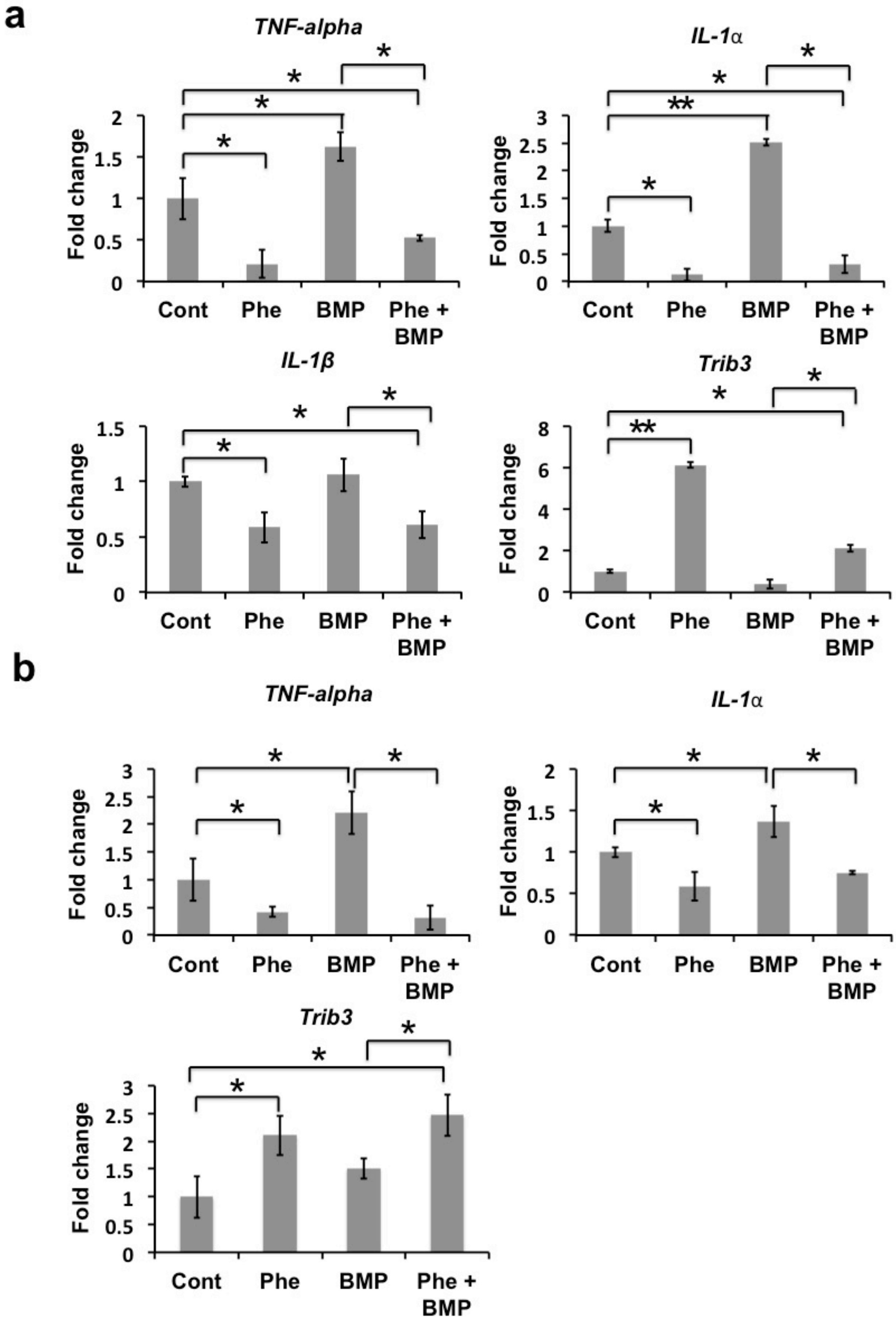

a
TNF-alpha
IL-1α
IL-1β
Trib3
b
TNF-alpha
IL-1α
Trib3
Fold change
Cont
Phe
BMP
Phe + BMP

Supplementary Figure 3: Phenamil (20 μM) inhibited high doses BMP2 (500 ng/mL)-induced inflammatory gene expression in NIH3T3 (a) and C2C12 cells (b). Phenamil treatment with or without BMP2 also enhanced Trib3 expression in both NIH3T3 (a) and C2C12 cells (b). Data presented as means ± SD (n=3/group); $*p < 0.05$, $**p < 0.01$.

.

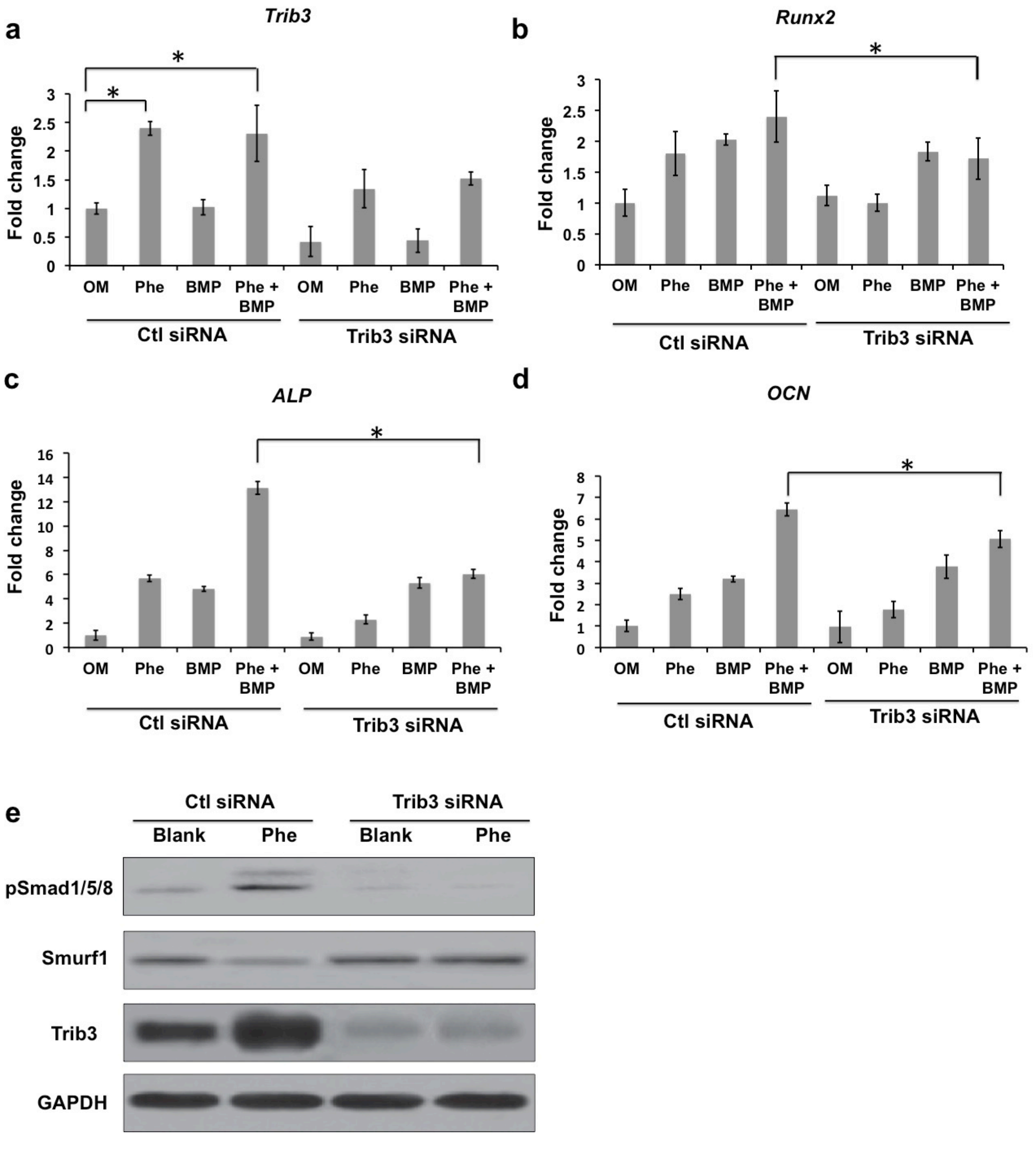

Supplementary Figure 4: (a-d) Knockdown Trib3 (a) blocked the synergistic effects of phenamil (20 μM) on BMP2 (100 ng/mL)-induced osteogenic genes: *Runx2* (b), *ALP* (c), *OCN* (d) in hBMSCs. (e) Western-blot assay indicated that Trib3 knockdown in hBMSCs reversed

phenamil-mediated BMP signaling that was stimulated by the enhanced Trib3 to stabilize pSmad1/5/8 *via* inhibiting Smurf-1. Data presented as means ± SD (n=3/group); *$p < 0.05$. Uncropped western blot gels are displayed in Supplementary Figure 6.

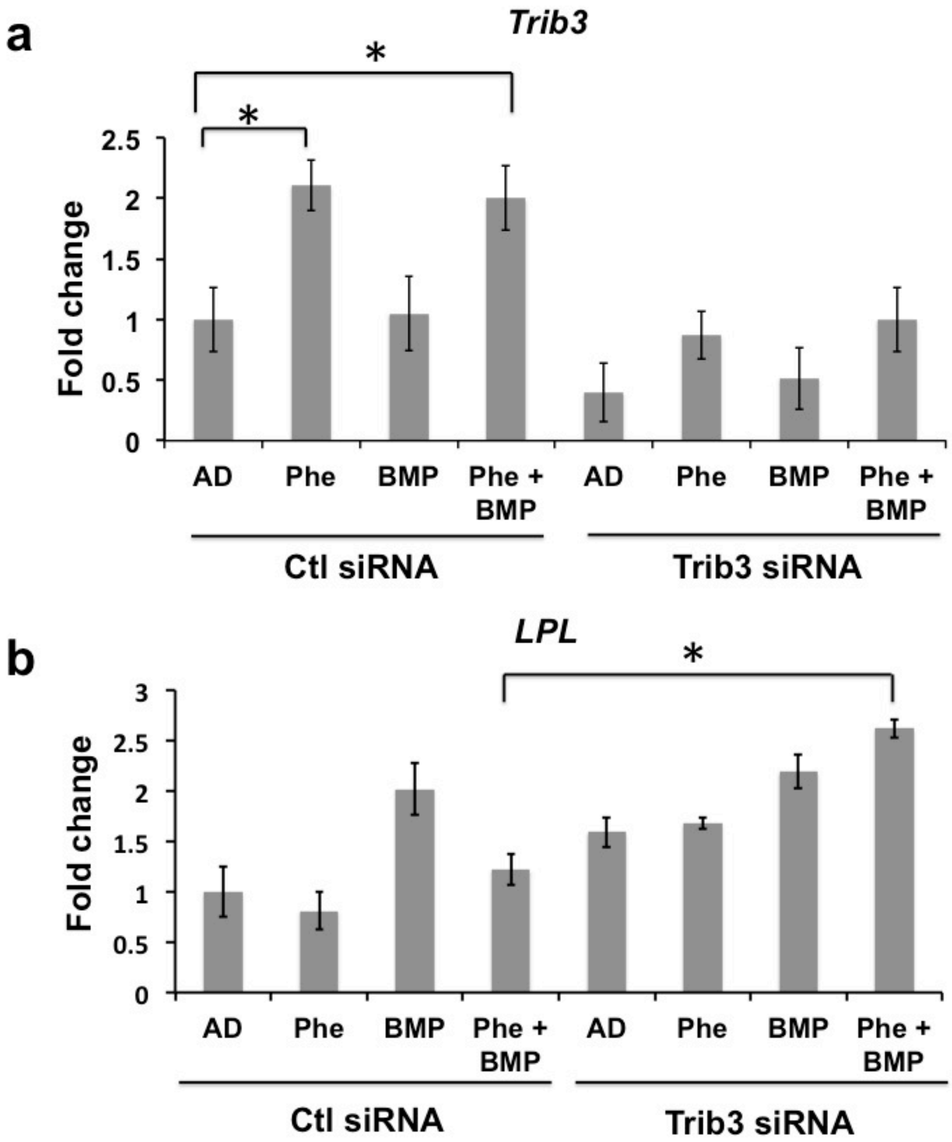

Supplementary Figure 5: (a,b) Trib3 knockdown (a) attenuated phenamil (10 µM)-mediated inhibitory BMP2 (300 ng/mL)-induced adipogenic genes: *LPL* (b) in hBMSCs. Data presented as means ± SD (n=3/group); *$p < 0.05$.

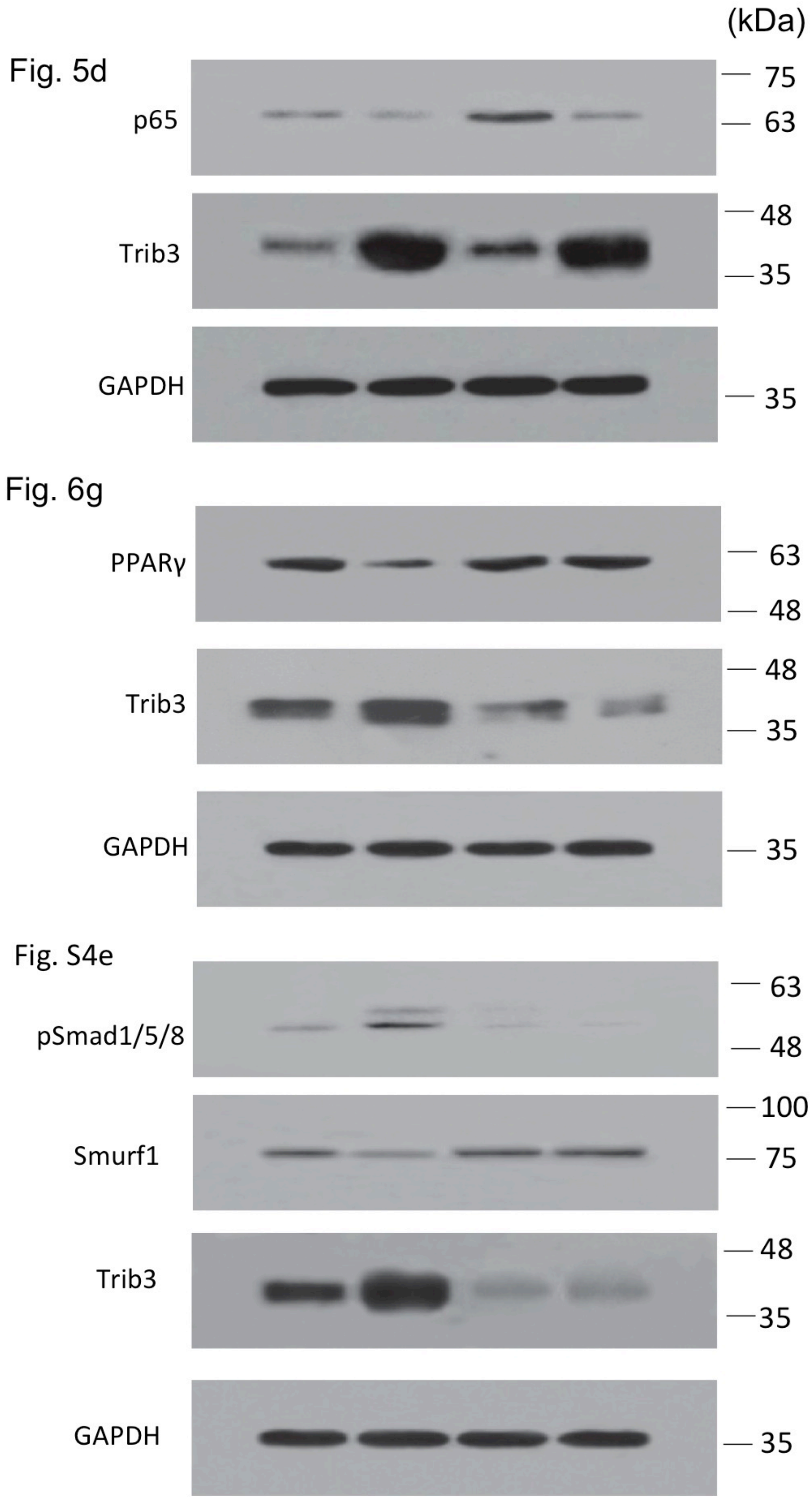

Supplementary Figure 6: Uncropped western-blot images related to Figure 5d, Figure 6g and Supplementary Figure 4e.

Supplementary Table 1: Primer sequences listed for real-time PCR assay.

Gene	Primer (Forward)	Primer (Reverse)
Runx2 (h)	TGGTTACTGTCATGGCGGGTA	TCTCAGATCGTTGAACCTTGCTA
ALP (h)	ACTGGTACTCAGACAACGAGAT	ACGTCAATGTCCCTGATGTTATG
Osteocalcin (h)	CACTCCTCGCCCTATTGGC	CCCTCCTGCTTGGACACAAAG
PPARγ (h)	GGGATCAGCTCCGTGGATCT	TGCACTTTGGTACTCTTGAAGTT
LPL (h)	TCATTCCCGGAGTAGCAGAGT	GGCCACAAGTTTTGGCACC
Trib3 (h)	AAGCGGTTGGAGTTGGATGAC	CACGATCTGGAGCAGTAGGTG
GAPDH (h)	TGTGGGCATCAATGGATTTGG	ACACCATGTATTCCGGGTCAAT
IL-1 α (m)	CGAAGACTACAGTTCTGCCATT	GACGTTTCAGAGGTTCTCAGAG
IL-1β (m)	GAAATGCCACCTTTTGACAGTG	TGGATGCTCTCATCAGGACAG
IL-6 (m)	CCAAGAGGTGAGTGCTTCCC	CTGTTGTTCAGACTCTCTCCCT
ALP (m)	AATGAGGTCACATCCATCCTG	CACCCGAGTGGTAGTCACAA
TNF-α (m)	CCCTCACACTCAGATCATCTTCT	GCTACGACGTGGGCTACAG
Trib3 (m)	GCTAGAGATCTGAGCCGGGTA	AAGAGAGCCTGGCAAGAGG
GAPDH (m)	TGTGTCCGTCGTGGATCTGA	CCTGCTTCACCACCTTCTTGA
